# 伴ZEB2-JAK2阳性Ph样急性B淋巴细胞白血病1例

**DOI:** 10.3760/cma.j.issn.0253-2727.2023.07.017

**Published:** 2023-07

**Authors:** 凯 沈, 曼 王, 德媛 胡, 虞莎 郭, 苏宁 陈

**Affiliations:** 苏州大学附属第一医院血液科，苏州 215006 Department of Hematology, the First Affiliated Hospital of Soochow University, Suzhou 215006, China

患者，男，42岁，因“乏力伴呼吸困难10余天”于2021年11月11日至当地医院就诊。患者既往体健，无特殊病史。查体：神志清，精神较差，贫血貌，全身皮肤黏膜无黄染，无瘀点、瘀斑，双肺呼吸音粗，未闻及明显干湿啰音，心律齐，各瓣膜未闻及病理性杂音，腹软，无压痛、反跳痛，肝脾未触及，双下肢无水肿。入院后查血常规：WBC 147.7×10^9^/L，HGB 27 g/L，PLT 18×10^9^/L。骨髓象：增生活跃，粒系、红系增生受抑，全片见巨核细胞20个。淋巴细胞占98.5％，其中原始及幼稚淋巴细胞占97.5％，其形态特点：胞体大小不等，核圆形或类圆形，部分细胞可见核切迹，核染色质细腻，核仁清晰或隐匿，胞质量少，染天蓝色。细胞化学染色：MPO：阴性；PAS：7％阳性（细颗粒状）；NBE：阴性。血液肿瘤免疫分型：在CD45/SSC点图上设门分析，原始向CD45阴性延伸的分布区域可见异常细胞群体，约占有核细胞的96％，主要表达HLA-DR、CD10、CD19、CD22、CD34、CD38、CD58、CD123、cCD79a、TdT。染色体核型：46,XY,?t（14;18）（q32;q21）,−16,+mar,inc[2]/46,XY[18]。白血病43种融合基因筛查定性检测：阴性。急性淋巴细胞白血病（ALL）相关46种基因突变分析：检测到PAX5基因移码突变（p.Arg199GlysTer45，48.3％）。明确诊断为急性B淋巴细胞白血病（B-ALL）。2021年11月17日行VDCP方案化疗，具体为：长春地辛6 mg第1、8、15天，伊达比星20 mg第1、8天，泼尼松30 mg第1～28天，环磷酰胺1.6 g第1、18天。2021年12月7日复查骨髓示形态学完全缓解（CR），微小残留病（MRD）：1.45％，染色体：46,XY,add（5）（q31）,?t（14;18）（q32;q21）,−16,add（16）（p13）,+mar,inc[2]/46,XY[18]。2021年12月23日起行Hyper-CVAD A方案巩固化疗，2022年1月26日于我院复查骨髓发现87％的原始幼稚细胞，提示患者B-ALL复发，同时加做转录组测序（RNA-seq）发现了ZEB2-JAK2融合基因。2022年1月31日行VP方案再诱导化疗，具体为：长春地辛4 mg第1、8天，地塞米松10 mg 第1～14天，同时入组了ssCART-19临床试验（U01S0152201）。2022年2月16日复查骨髓：增生明显活跃，原始幼稚细胞占65.5％。该患者明确诊断为伴有JAK2重排的难治复发性Ph样B-ALL。2022年2月21日行FC方案预处理，具体为：环磷酰胺700 mg第1～3天，氟达拉滨70 mg第1～3天。2022年3月3日骨髓象：增生减低，原始幼稚细胞占40％，MRD：42.54％。2022年3月4日回输CD19^+^CAR-T细胞，总量为15×10^5^/kg。回输第9天出现了细胞因子释放综合征3级反应，予地塞米松、芦可替尼、输注血制品等对症处理后好转。CAR-T细胞回输28 d后复查骨髓示形态学CR，MRD：10.4％。患者因经济原因后续没有选择行造血干细胞移植治疗。2022年5月6日患者外周血涂片发现88％的原始幼稚细胞，流式细胞术提示原始异常细胞约占93％，患者本病再次复发，随访至2022年6月30日，患者于当地医院治疗中，本病仍处未缓解状态。

讨论：我们首次在Ph样B-ALL患者中新发现了ZEB2-JAK2融合基因，该患者对常规化疗反应不佳，经CAR-T细胞治疗后本病缓解，表明CAR-T细胞免疫治疗可能是此类患者可行的选择。

